# Re-Emergence of Circulation of Seasonal Influenza during COVID-19 Pandemic in Russia and Receptor Specificity of New and Dominant Clade 3C.2a1b.2a.2 A(H3N2) Viruses in 2021–2022

**DOI:** 10.3390/pathogens11111388

**Published:** 2022-11-21

**Authors:** Natalia P. Kolosova, Tatiana N. Ilyicheva, Vasily V. Unguryan, Alexey V. Danilenko, Svetlana V. Svyatchenko, Galina S. Onhonova, Natalia I. Goncharova, Maksim N. Kosenko, Andrey S. Gudymo, Vasiliy Y. Marchenko, Alexander N. Shvalov, Ivan M. Susloparov, Tatiana V. Tregubchak, Elena V. Gavrilova, Rinat A. Maksyutov, Alexander B. Ryzhikov

**Affiliations:** 1 State Research Centre of Virology and Biotechnology “Vector”, Rospotrebnadzor, Koltsovo, Novosibirsk 630559, Russia; 2 Department of Physics, Novosibirsk State University, Novosibirsk 630090, Russia

**Keywords:** seasonal influenza virus, COVID-19 pandemic influence, monitoring, molecular modeling, A(H3N2) clade 3C.2a1b.2a.2, receptor specificity

## Abstract

The circulation of seasonal influenza in 2020–2021 around the world was drastically reduced after the start of the COVID-19 pandemic and the implementation of mitigation strategies. The influenza virus circulation reemerged in 2021–2022 with the global spread of the new genetic clade 3C.2a1b.2a.2 of A(H3N2) viruses. The purpose of this study was to characterize influenza viruses in the 2021–2022 season in Russia and to analyze the receptor specificity properties of the 3C.2a1b.2a.2 A(H3N2) viruses. Clinical influenza samples were collected at the local Sanitary-and-Epidemiological Centers of Rospotrebnadzor. Whole genome sequencing was performed using NGS. The receptor specificity of hemagglutinin was evaluated using molecular modeling and bio-layer interferometry. Clinical samples from 854 cases of influenza A and B were studied; A(H3N2) viruses were in the majority of the samples. All genetically studied A(H3N2) viruses belonged to the new genetic clade 3C.2a1b.2a.2. Molecular modeling analysis suggested a higher affinity of hemagglutinin of 3C.2a1b.2a.2. A(H3N2) viruses to the α2,6 human receptor. In vitro analysis using a trisaccharide 6’-Sialyl-N-acetyllactosamine receptor analog did not resolve the differences in the receptor specificity of 3C.2a1b.2a.2 clade viruses from viruses belonging to the 3C.2a1b.2a.1 clade. Further investigation of the A(H3N2) viruses is required for the evaluation of their possible adaptive advantages. Constant monitoring and characterization of influenza are critical for epidemiological analysis.

## 1. Introduction 

At the end of 2019, a new coronavirus (SARS-CoV-2) appeared in China where it began to spread around the world. This led to the COVID-19 pandemic, which is still ongoing. Since February–March 2020, the circulation of influenza A and B viruses, respiratory syncytial viruses, paramyxoviruses, seasonal coronaviruses, metapneumoviruses, and adenoviruses have sharply decreased. However, at the same time, the circulation of enteroviruses/rhinoviruses continued with regional differences in intensity during the seasonal epidemic [1]. In the 2020−2021 season, an unprecedented low sporadic influenza circulation was observed. The epidemic threshold was not reached in any country, and the detection of seasonal influenza was even lower than during normal summer months [2]. 

One of the reasons for the very low incidence of influenza during the COVID-19 pandemic was the use of individual and community measures to protect public health around the world such as travel restrictions, use of respiratory protection, and social distancing [3]. However, in some countries, anti-epidemic measures were not applied as harshly as in Europe, North America, and some countries in Southeast Asia, but despite this, influenza circulation there was also extremely low. Viral interference could be another important factor for the phenomenon. Accumulating evidence shows that one respiratory virus can block infection by another virus by stimulating antiviral defenses [4,5]. Analysis of co-infections with several respiratory viruses, such as rhinovirus and influenza A virus, showed that co-infections are much less common than would be expected if the event occurred by chance [6]. Apparently, due to the lower reproductive number (R0) of seasonal influenza viruses compared to SARS-CoV-2, which could be partially due to already present herd immunity against influenza, stringent epidemic control measures were much more effective in reducing the circulation of influenza viruses in 2020–2021 [7]. 

Due to the low circulation of influenza, a difficult situation arose in regard to the decision-making on the composition of the seasonal vaccine since much fewer influenza viruses were genetically characterized in the world and, in the period from 2020–2021, the emergence and spread of new antigenic groups of influenza A (H3N2) and B/Victoria viruses were observed [8,9]. 

In the Northern Hemisphere, a substantial circulation of influenza reemerged in the 2021–2022 season with peak circulation at the beginning of January at the end of the COVID-19 Delta variant (Lineage Pango: B.1.617.2) wave (Worldometers.info/coronavirus/ (accessed on 1 September 2022), Next Strain/ncov (accessed on 1 September 2022) and the start of Omicron variant (Lineage Pango: B.1.1.529) wave (Worldometers.info/coronavirus/ (accessed on 1 September 2022), Next Strain/ncov (accessed on 1 September 2022)). In the 2021–2022 season, globally in the Northern Hemisphere, the influenza A(H3N2) virus of the new clade 3C.2a1b.2a.2 (antigenically different from the vaccine strain of the Northern Hemisphere) prevailed in circulation with a small proportion of influenza A(H1N1)pdm09 and influenza B circulation (with the exception of China, where the influenza B and A(H3N2) clade 3C.2a1b.2a.1 dominated) [10]. 

Hemagglutinin of clade 3C.2a1b.2a.2 viruses have amino acid substitutions Y159N, T160I, and D190N [9], which can potentially affect receptor specificity [Flusurver]. This could be one of the factors facilitating the spread of this clade amidst the SARS-CoV-2 circulation and pandemic response actions.

For influenza virus entry into the host cell, hemagglutinins interact with the oligosaccharides, terminated by sialic acid. Alpha 2-6 linked sialic acid oligosaccharides are spread on the membrane surfaces of the human upper respiratory tract epithelial cells, being part of glycolipids and glycoproteins [11]. Influenza virus hemagglutinin receptor specificity and affinity play an important role in the transmissibility and infectivity of the virus [12]. 

Significant changes in the receptor-binding site of influenza A(H3N2) hemagglutinin accumulate in the process of evolution. This can lead to changes in virological properties and, possibly, to changes in the epidemiological impact [13]. For the evaluation of influenza virus receptor specificity and affinity, a variety of methods have been employed including in vitro methods (such as bio-layer interferometry and ELISA) and molecular modeling methods, which include a wide array of approaches for analysis of the interaction of receptor binding domain of hemagglutinin with receptor analogs. A recent in vitro study of the HA receptor specificity of 3C.2a1b.2a.2 A(H3N2) viruses indicated a change in receptor structure preference compared to viruses from the 3C.2a1b.2a.1 clade [14]. Analysis of the receptor specificity of influenza viruses is important for the understanding of virus evolution, and adaptation and evaluation of virus pandemic potential.

The unpredictability of the course of the influenza seasons, the constant threat of an influenza epidemic, and the additional risk of co-circulation of influenza viruses and SARS-CoV-2 have highlighted the need for ongoing close monitoring of influenza. Data on the genetic variability of circulating strains, their difference from vaccine strains, and characterization of drug susceptibility and virulence properties are necessary for epidemiological analysis and for providing information on the circulating strains of influenza virus in Russia to the WHO for the selection of influenza vaccine strains. 

The main aim of this work was to characterize seasonal influenza viruses in the 2021–2022 season in Russia, which followed the 2020–2021 season that had a near absence of influenza circulation. In addition to the genetic and virological characterization of circulated viruses, another aim of the study was the investigation of the receptor specificity properties of the 3C.2a1b.2a.2 A(H3N2) viruses which dominated circulation in the 2021–2022 season.

In Russia, the 2021–2022 influenza season was characterized by an almost complete dominance of 3C.2a1b.2a.2 A(H3N2) influenza viruses and sporadic circulation of subtype A(H1N1)pdm09 and influenza B/Victoria viruses. Molecular modeling using molecular dynamics methods showed that the hemagglutinin of the 3C.2a1b.2a.2 A(H3N2) viruses binds to the human alpha 2,6 sialic acid receptor analog with more rigidity in the complex and the molecular docking method showed a lower docking score for the HA, thus suggesting the increase in affinity to a human-type receptor, which may be associated with higher infectivity and transmissibility.

## 2. Material and Methods 

### 2.1. Sample Preparation and Influenza Diagnostics

The study of clinical material (nasopharyngeal swabs) and autopsy materials were approved by the Ethics Committee IRB 00001360 affiliated with the Federal Budgetary Research Institution State Research Center of Virology and Biotechnology “Vector”, Rospotrebnadzor (SRC VB “Vector”) (http://www.vector.nsc.ru/eticheskiy-komitet/ (accessed on 1 September 2022)). 

Samples were collected at the local Sanitary-and-Epidemiological Centers of the Federal Service for Surveillance of Consumer Rights Protection and Human Wellbeing (Rospotrebnadzor, https://rospotrebnadzor.ru/region/structure/str_fguz.php (accessed on 1 September 2022)) after obtaining written, informed consent from the patients or their close relatives in accordance with the regulations of the Russian Federation. PCR-based diagnostics of the original material for influenza virus RNA were conducted in local laboratories, and then all the positive samples were sent to the SRC VB “Vector”, Rospotrebnadzor. All samples received in the SRC VB “Vector”, Rospotrebnadzor were retested using diagnostic PCR. Viruses were isolated in MDCK cells [15]. Diagnostic real-time PCR for detecting influenza A and B viruses was performed using the reagent kits “AmpliSense Influenza virus A/B-FL”, “AmpliSense Influenza virus H1N1pdm2009-FL”, and “AmpliSense Influenza virus H3N2-FL” manufactured by the Central Research Institute of Epidemiology of the Federal Service for Surveillance of Consumer Rights Protection and Human Wellbeing (Moscow, Russia). RNA was isolated with the “RIBO-prep” kit and cDNA was synthesized using the “RevertaL” kit. 

### 2.2. Hemagglutination Inhibition Assay 

The antigenic properties of isolated influenza A(H3N2) viruses were studied by a hemagglutination inhibition assay (HIA) with reference ferret antisera raised against egg-propagated vaccine strains recommended for the 2021–2022 (A/Cambodia/e0826360/2020) and 2022–2023 (A/Darwin/9/2021) influenza seasons in the Northern Hemisphere and cell culture-propagated strain A/Stockholm/5/2021, which belongs to the same clade as strain A/Darwin/6/2021, from the 2022–2023 cell-based vaccine for the Northern Hemisphere. A HIA for A(H3N2) viruses was performed using a turkey red blood cell (RBC) suspension with 20nM oseltamivir. Isolated influenza B/Victoria viruses were tested by a HIA with ferret antisera raised against egg- and cell-propagated variants of the B/Victoria vaccine strains recommended for the 2021–2022 (B/Washington/02/2019) and 2022–2023 (B/Austria/1359417/2021) Northern Hemisphere influenza seasons. A HIA for B/Victoria viruses was performed using turkey RBC. All reference reagents (ferret antisera and corresponding viruses) were kindly provided by the Worldwide Influenza Centre at the Francis Crick Institute, London (WHO Collaborating Centre for Reference and Research on Influenza) [15].

### 2.3. Phenotypic Analysis of Neuraminidase Inhibition by Oseltamivir and Zanamivir

Analysis of the phenotypic inhibition of influenza virus neuraminidase by oseltamivir and zanamivir was performed by a fluorescent neuraminidase inhibition assay with 2-(4-methylumbelliferyl)-a-D-N-acetylneuraminic acid (MUNANA) substrate [16].

### 2.4. Analysis of Herd Immunity

Blood samples were collected from donors who provided written informed consent on the condition of anonymity. Sera collection from healthy donors and the hemagglutination inhibition (HI) test were performed as previously described [17]. A/Victoria/2570/2019 (H1N1)pdm09, A/Cambodia/e0826360/2020 (H3N2), B/Washington/02/2019 (B/Victoria lineage), and B/Phuket/3073/2013 (B/Yamagata lineage) influenza viruses were kindly provided by the WHO Collaborating Center in London, UK. 

### 2.5. Sequence Analysis of Influenza Viruses 

Sequencing was carried out at the SRC VB “Vector”, Rospotrebnadzor. To determine the nucleotide sequences of viral genes and genomes, viral RNA was isolated using the RIBO-sorb RNA/DNA Extraction Kit (InterLabService, Moscow, Russia) according to the manufacturer’s instructions. Reverse transcription was carried out with a mixture of primers (Uni12, Uni12.4, and Uni13) [18] for type A influenza virus samples and Uni11 primer for type B influenza virus samples using the OT-M-MuLV-RH reagent kit (LLC BIOLABMIX, Russia). PCR amplification of cDNA was performed using the BioMaster LR HS-Taq PCR kit (2×) (OOO BIOLABMIKS, Russia) according to the manufacturer’s instructions and previously described protocols with modifications [18,19]. Detailed protocols are available upon request. Deep sequencing of amplicons covering complete genomes was performed on an Illumina MiSeq using the MiSeq reagent kit v3 (Illumina, San Diego, CA, USA). The full-length genomes were assembled by the alignment of reads to known references with bwa-0.7.15 [20]. The obtained nucleotide sequences were deposited in the Global Initiative on Sharing All Influenza Data (GISAID) database. Phylogenetic analysis was performed using the maximum likelihood method based on the Hasegawa–Kishino–Yano model with 1000 bootstrap replications using MEGA 6.0 software (http://www.megasoftware.net/, accessed on 1 September 2022) [21]. For comparison, sequences of strains deposited in GISAID were used. Amino acid sequences of the isolated strains’ genomes were analyzed using FluSurver (http://flusurver.bii.a-star.edu.sg, accessed on 1 September 2022).

### 2.6. Molecular Modeling of HA Receptor Specificity to Human Type Alpha 2,6 Sialoside Receptor

The molecular docking method was applied to estimate the affinity of complexes of HA with trisaccharide receptor analog 6′-sialyl-N-acetyllactosamine using the software Autodock Vina [22]. Because the crystal structures of the complexes are unknown, we applied the ensemble docking approach [23], starting from homology-modeled glycoproteins and receiving the configuration ensembles from the molecular dynamics trajectories. Initial complexes were modeled by the comparative modeling method with MODELLER [24,25]. For comparative modeling, the structure (complex HA-receptor analog) with the code 6bkt from Protein Data Bank was used as a template. The receptor analog was transferred from the template into the initial models as a rigid body.

To account for possible configurations of glycoproteins and the receptor analog, the molecular dynamics trajectories were recorded for the complexes. In the process of system preparation, saccharides related to the glycosylation sites were transferred from the template crystal structure and imposed, then linked with corresponding protein residues. All glycans and proteins were parametrized with GLYCAM06 [26] and FF14SB Amber [27] force fields, correspondingly. Every complex was solvated in a TIP3P rectangular water box; the size of the box was defined to provide a minimum distance of 10 A between the boundaries and the solute. Na and Cl ions were added to the 0.15 M concentration to mimic physiological conditions. These procedures were performed using the LEaP module of AmberTools22 [27].

Molecular Dynamics (MD) simulations were carried out in NAMD 2.14 [28]. Prior to the main simulation, equilibration was executed in several cycles: (1) water, ions, and hydrogens, (2) protein sidechain, and (3) all atoms were equilibrated with 10000 energy minimization steps before each equilibration cycle. The rest of the atoms in the equilibration process were constrained. The time step of the simulations was set to 2 femtoseconds. The temperature, 310 K, was maintained with Langevin dynamics and the pressure, 1 Atm, was controlled with the hybrid Nosé–Hoover Langevin piston method [29]. The cutoff radius was set to 10 A for the direct count of electrostatics and long-range electrostatics were treated with the Particle Mesh Evald method in conjugation with periodic boundary conditions. 1-4 electrostatic and VdW interactions were scaled by standard AMBER values SCEE = 1.2 and SCNB = 2.0. The frames were saved every 3 picoseconds. The full simulation continued for 160 ns; the last 100 ns was used for the following analysis.

To choose the representative frames for molecular docking estimation, we used principal component analysis [30]. The non-hydrogen atom coordinates of residues with numbers 98, 131, 135-137, 153, 155, 156, 183, 186, 187, 189, 190, 193, 194, 222, and 225-228, which are located in close position to the crystal structure of the receptor analog, were used for the principal component calculations. The covariance matrix was calculated and diagonalized, then the atoms’ positions in all frames were projected onto the new collective space. For the docking estimation, we selected 100 frames, corresponding to the maximums along the first 25 principal components. The principal component analysis was performed in the CPPTRAJ module of AmberTools22 [31]. 

Molecular docking was performed in a box measuring 30 × 30 × 30 A, sized around the receptor analog center mass position with Autodock Vina 1.2.3 version. The glycoside torsion angles were frizzed in the docking process, as was recommended [32], because of stereoelectronic effects [33] influencing the relative orientations of the pyranose rings. The structures for the proteins and glycoside torsion angles for the receptor analog were taken from the selected frames in the previous step. The scripts from the ADFR software suite [34] were used for the preparation of structures for molecular docking. The distance between grid points was set to the standard value of 0.375 A, and the parameter exhaustiveness equal to 64 was used.

### 2.7. Preparation of Inactivated Viral Stocks for Receptor-Binding Assay

Reference influenza A (H3N2) viruses and several seasonal influenza A(H3N2) viruses isolated during the 2021–2022 flu season in Russia were analyzed in the receptor-binding assay. The isolation of seasonal influenza viruses from human nasopharyngeal swabs or organ fragments (trachea, bronchi, or lungs) was performed in MDCK cell cultures [15]. Reference viruses used in the receptor-binding assay (A/Cambodia/925256/2020, A/Hong Kong/45/2019, A/Cambodia/e0826360/2020, and A/Stockholm/5/2021, A/Darwin/09/2021) were kindly provided by the Worldwide Influenza Centre at the Francis Crick Institute, London (WHO Collaborating Centre for Reference and Research on Influenza). Viruses isolated from the original clinical specimens and reference viruses were propagated in MDCK cells. Following the completion of the cytopathic effect and one freeze-thaw cycle, the culture medium was harvested and centrifuged at 5000 rpm for 10 min. Collected virus-containing supernatants were inactivated with β-propiolactone. For this, one part of 2% β-propiolactone solution in chilled distilled water was added to 38 parts of the virus-containing medium. Then, the mixture was incubated in a water bath at 37 ℃ for 2 h with manual stirring every 15 min. Virus inactivation was considered successful if there were no signs of cytopathogenic effect during three consecutive blind passages in MDCK cells. The passage history of viral stocks used in the receptor-binding assay was as follows: A/Stockholm/5/2021 (SIAT3/MDCK1); A/Yekaterinburg/10-01V/2021 (MDCK2); A/YANAO/08-01V/2021 (MDCK2); A/Cambodia/925256/2020 (SIAT4/MDCK2); A/Cambodia/e0826360/2020 (E5/E2/MDCK1); A/Hong Kong/45/2019 (Cx/MDCK2); and A/Darwin/09/2021 (E3/E2/MDCK1).

### 2.8. Virus Purification for Receptor-Binding Assay

A medium containing inactivated virus was centrifugated at low speed to remove large debris and was then filtered using a membrane filter (0.45 µm). Virus particles were next pelleted by ultracentrifugation and resuspended in phosphate-buffered saline (PBS). Size-exclusion chromatography was performed using Sepharose CL-4B resins (GE Healthcare). Finally, influenza A virions were concentrated using Amicon^®^ Ultra-15 Centrifuge Filters Ultracell^®^ 100KDa (Merck Millipore). The concentrations of purified viruses were determined by HA assays [35]. 

### 2.9. Receptor Binding Assay

The kinetics of the interaction of influenza virions with biotinylated trisaccharide 6’-Sialyl-N-acetyllactosamine receptor analog 0997-BP (Lectinity) was measured by biolayer interferometry using an Octet RED96e (ForteBio). The sugars were loaded onto streptavidin biosensors at a concentration of 0.5 μg/mL and then viruses at 10-100 nM were added. To inhibit neuraminidase, oseltamivir (20 nmol/L) was added to viruses. Obtained data were analyzed by Octet software. 

## 3. Results

### 3.1. Genetic and Virological Analysis of Circulating Viruses in Russia in 2020–2022

During the analysis of seasonal influenza in 2020–2021, sporadic circulation of influenza B viruses was detected. Only six samples were received, in which the influenza virus was detected using reference diagnostics. All analyzed viruses were type B influenza. Genetic analysis of the six influenza B viruses showed that all the studied viruses belonged to the B/Victoria genetic lineage, V1A.3 clade, with a deletion of three amino acids in HA1 (162-164) and to the V1A.3a subclade with characteristic amino acid substitutions in HA - N150K, G184E, N197D, and R279K, in which, two groups, 3a1 and 3a2, were distinguished. Two of the studied viruses belonged to the group 3a1, with characteristic substitutions in HA V220M and P241Q (B/Yekaterinburg/3291V/202 and, B/Yekaterinburg/3292V/2020), and four of the studied viruses belonged to group 3a2, with characteristic substitutions A127T, P144L, and K203R (B/Rostov/340V/2021, B/Tyumen/343V/2021, B/Tyumen/3432V/2021, and B/Zabaykalsky Krai/349V/2021) [36].

During the analysis of seasonal influenza in 2021–2022 in Russia, SRC VB Vector samples from 854 influenza cases were confirmed for the presence of influenza virus genetic material by reference diagnostics. Influenza viruses of A(H3N2), A(H1N1)pdm09, and B subtypes were identified in 97.8%, 0.3%, and 1.9% of confirmed cases, respectively. The majority of analyzed influenza cases (72%) were in children under 17 years of age and 20% of studied cases were found in children under 5 years of age. The majority of received samples were from influenza cases that occurred from November 2021 to the end of January 2022. A total of 12 fatal cases were confirmed, among which, 11 cases of influenza A(H3N2) and 1 case of influenza B and SARS-CoV-2 coinfection were identified. During the 2021–2022 season, 215 influenza A(H3N2) viruses and 3 influenza B/Victoria viruses were isolated in the MDCK cell cultures. NGS was performed using isolates and primary material for a representative sample of viruses including 90 A(H3N2) viruses, 2 A(H1N1)pdm09 viruses, and 7 influenza B/Victoria viruses. 

**Figure 1:**
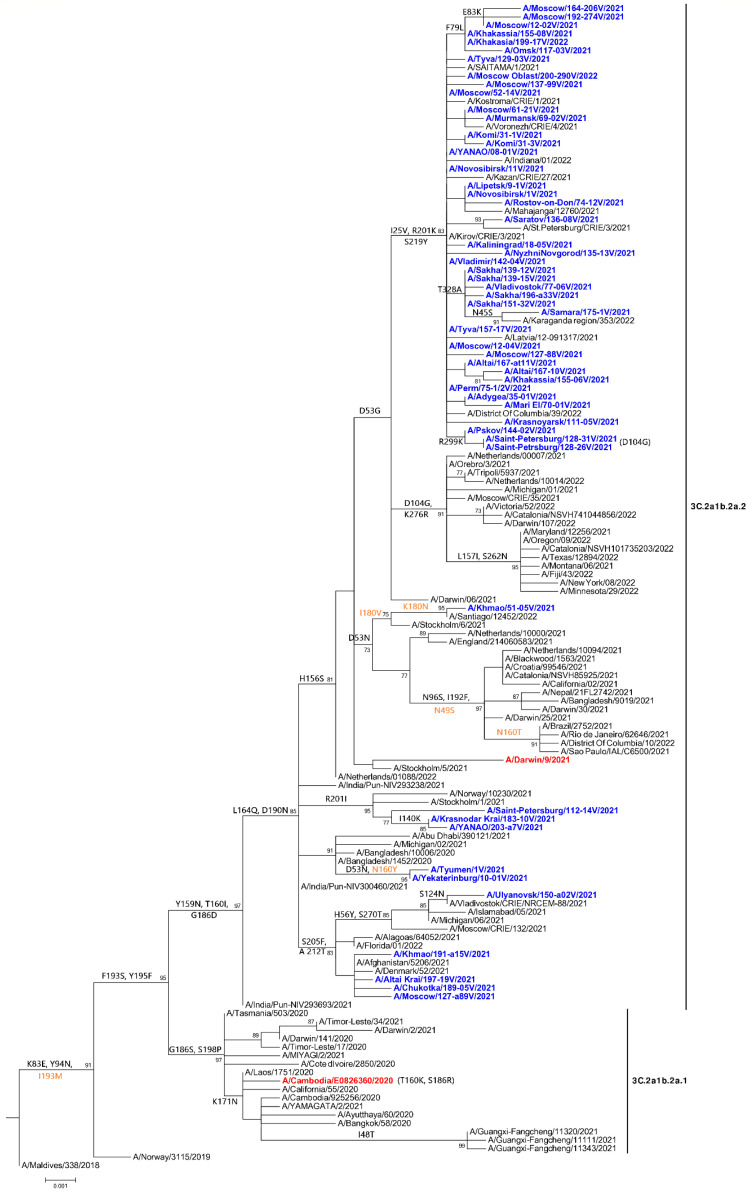
The phylogenetic tree for the HA of a representative set of A(H3N2) influenza viruses analyzed in this study, Russia, influenza season 2021–2022. Viruses isolated in Russia in the 2021–2022 epidemic season are indicated in blue. Vaccine strains are indicated in red. Amino acid substitutions in HA1 are in black and amino acid substitutions in HA2 are in brown.

Phylogenetic and molecular genetic analysis showed that all studied 90 influenza A(H3N2) viruses belonged to the 3C.2a1b.2a.2 clade (2a2) (A/Bangladesh/10006/2020 is the reference strain of the group 2a2) (Figure 1).

Mutations in the HA that characterize this group include the Y159N and T160I substitutions (resulting in the loss of a glycosylation site) and the L164Q, G186D, and D190N substitutions [10]. Most of the studied A(H3N2) viruses (89%, 80 out of 90) had additional mutations in HA: D53G and H156S and a group of mutations I25V, R201K, and S219Y, characteristic of viruses circulating in Russia, compared to the vaccine strain A/Cambodia/E0826360/2020. The analysis of A(H3N2) viruses using Flusurver revealed that the amino acid substitutions Y159N, T160I, and D190N in HA, which is characteristic of 2a2 clade [9] and is present in all identified A(H3N2) viruses in Russia, may be associated with changes in receptor specificity.

Among the 11 analyzed cases of influenza A(H3N2) with a fatal outcome, 7 had mutations I25V, R201K, and S219Y, characteristic of circulating A(H3N2) strains in Russia. Additional mutations that may be associated with increased pathogenicity were not found in viruses from the fatal cases. Among the 11 fatal cases, there were no vaccinated cases and 7 of the 11 cases belonged to a risk group as determined by age.

In order to assess the antigenic properties of isolated influenza A(H3N2) viruses, 44 isolates were analyzed in HIA with ferret antisera raised against vaccine and reference influenza A(H3N2) viruses. Serum raised against the egg-propagated 2021–2022 Northern Hemisphere vaccine strain, A/Cambodia/e0826360/2020 (clade 3C.2a1b.2a.1), recognized only 25% of 44 analyzed isolates within 4-fold of the homologous serum titer. Serum titers were 8-times lower than homologous titers for 70.4% of tested isolates. For 4.6% of tested isolates, serum titers were 16-32 times lower in comparison with homologous titers. At the same time, all studied isolates reacted well with ferret serum raised against the egg-propagated 2022–2023 vaccine strain A/Darwin/9/2021 (clade 3C.2a1b.2a.2): reverse serum HI titers in assays with isolated viruses differed from the homologous serum titer no more than two times. A subset of tested A(H3N2) isolates (N=38) were additionally analyzed with serum against the cell-propagated reference strain A/Stockholm/5/2021 from the same clade as A/Darwin/9/2021 (clade 3C.2a1b.2a.2). The anti-A/Stockholm/5/2021 serum recognized 37 isolates at titers no more than two times less than homologous titer. The HI titer in assays with one isolate was four times less than the homologous titer.

In Russia, genetic analysis was carried out only for two influenza A(H1N1)pdm09 viruses on the basis of the SRC VB “Vector”, Rospotrebnadzor. Phylogenetic analysis of the two influenza A(H1N1)pdm09 viruses showed that they belonged to clade 6B.1A.5a with characteristic substitutions in HA1 N129D, T185I, and N260D. At the same time, one of the viruses, A/Moscow/154-161V/2021, belonged to subclade 5a1, with characteristic substitutions in HA D187A and Q189E, and one virus, A/Khmao/182-14V/2021, belonged to subclade 5a2 with characteristic substitutions in HA K130N, N156K and L161I [10].

Additional mutations have been identified in the A/Khmao/182-14V/2021 virus including A186T, Q189E, E224A, R259K, and K308R compared to A/Victoria/2570/2019.

Phylogenetic analysis showed that A/Khmao/182-14V/2021 belongs to a new diversified group of A(H1N1)pdm09 with A/Sydney/5/2021 as a candidate vaccine virus (CVV) reference strain [36].

The genetic analysis of seven influenza B viruses showed that all studied viruses belonged to the B/Victoria genetic lineage and belonged to subclade 1A.3a2 (with characteristic substitutions A127T, P144L, and K203R). The vaccine strain B/Austria/1359417/2021 for the 2022 season for the Southern Hemisphere also belonged to subclade 3a2. 

In one fatal case, a co-infection with the B/Volgograd/202-a104V/2021 influenza virus and the SARS-CoV-2 virus was detected. In the influenza virus B/Volgograd/202-a104V/2021, no mutations associated with increased pathogenicity were found. No viruses of the B/Yamagata genetic lineage were found. Two influenza B/Victoria isolates were tested in a HIA with reference antisera. Serum raised against the egg-propagated 2020–2021 Northern Hemisphere vaccine strain B/Washington/02/2019 (V1A.3) recognized both isolates at titers 16 times less than the homologous titer. Serum raised against the cell culture-propagated variant of the same strain recognized both isolates within 2-fold of the homologous titer. Sera raised against the egg and cell-propagated variants of the 2022–2023 Northern Hemisphere vaccine strain B/Austria/1359417/2021 (V1A.3a.2) recognized both isolates at titers equal to or more than the homologous titer. 

### 3.2. Drug Susceptibility

Genetic analysis showed that all studied viruses in 2020–2022, for which NA gene sequence was available (total 99), did not contain amino acid substitutions in the NA gene responsible for drug resistance to neuraminidase inhibitors (the list of the NA drug resistance markers is provided by a WHO expert working group on analysis of influenza antiviral susceptibility (AVWG)) [37]. Analysis of the PA gene of all studied viruses, for which the PA gene sequence was available (total 84), showed that there were no mutations in the PA gene associated with drug resistance to baloxavir (the list of the PA drug resistance markers is provided by the WHO AVWG) [37]. 

During the 2021–2022 influenza season, 84 influenza A(H3N2) and 3 influenza B/Victoria isolates were tested in a fluorescence-based neuraminidase inhibition assay in this study. All tested A(H3N2) and B/Victoria isolates were characterized by normal inhibition of neuraminidase activity by oseltamivir and zanamivir according to the WHO AVWG criteria [38].

### 3.3. Investigation of Herd Immunity

In September-November 2021, about 1344 blood serum samples were collected in five regions of Russia: in the south of the European part, in the Volga region, in the Urals, in Siberia, and in the Russian Far East. HI tests of all the samples with vaccine strains recommended by the WHO for the Northern Hemisphere 2021–2022 season showed that 55.4% of the samples were seronegative to all vaccine strains of influenza A and B viruses.

### 3.4. Receptor Specificity Analysis of A(H3N2) Using Molecular Modeling 

Comparative analysis of the HA-receptor interaction of A(H3N2) viruses, A/Cambodia/E0826360/2020 (clade 3C.2a1b.2a.1) and A/YANAO/08-01V/2021 (clade 3C.2a1b.2a.2), was performed using molecular modeling methods with trisaccharide receptor analog 6’-sialyl-N-acetyllactosamine. 

The amino acid sequence identity of the HA of the template (code 6bkt from Protein Data Bank), which was used for the homology modeling, was 96.4% and 94.8% with A/Cambodia/E0826360/2020 and A/YANAO/08-01V/2021 HA, correspondingly. The quality of the obtained models was evaluated by the calculation of the root mean square deviations of backbone carbon atoms (CA atoms) in the HA1 subunit between the template and the studied viruses. These values were 0.15 and 0.14 angstrom for A/Cambodia/E0826360/2020 and A/YANAO/08-01V/2021, respectively, indicating the good quality of the models and fitness of the template [39]. 

**Figure 2:**
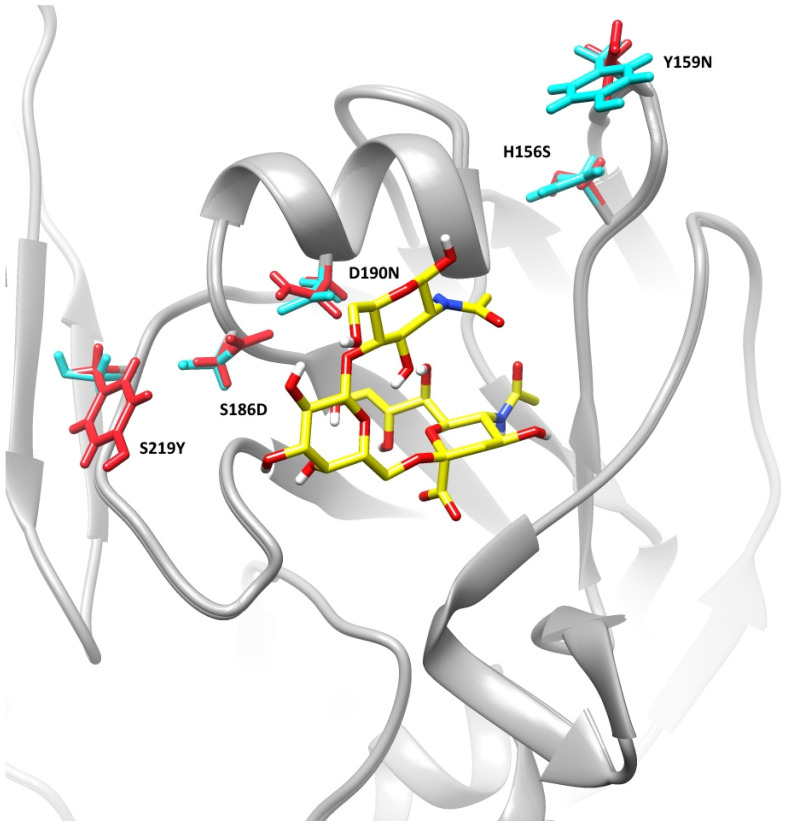
The receptor-binding site of homology modeled hemagglutinins. The blue and red colors highlight the substituted side chains and are related to A/Cambodia/E0826360/2020 and A/YANAO/08-01V/2021, correspondingly. The receptor analog is colored by atoms.

Analysis of the receptor binding site of homology-modeled hemagglutinins showed that the substitutions D190N and S186D (190-helix) and S219Y (220-loop) may induce the change of receptor affinity due to their close position to the crystal conformation of the receptor analog (Figure 2). The effect of the changes of amino acids in the receptor-binding site on the receptor-binding properties was further investigated using molecular dynamics and docking simulations.

The analysis of the molecular dynamics trajectories for the HA of the studied viruses (Figure S1) showed that only conformations close to the crystallographic pose are observed for A/YANAO/08-01V/2021. Such conformations were dominant for A/Cambodia/E0826360/2020, however, other conformations (Figures S1 and S2) were also present in the trajectory, indicating the more static behavior of the receptor analog in the complex with the HA of A/YANAO/08-01V/2021. The more rigid behavior of the receptor analog in the complex with A/YANAO/08-01V/2021 indicates that substitutions in the 220-loop and 190-helix may result in changes in the interaction profiles between the receptor analog and those close to the helix region of HA. These changes can promote the stabilization of receptor analog conformation and increase receptor affinity.

**Figure 3:**
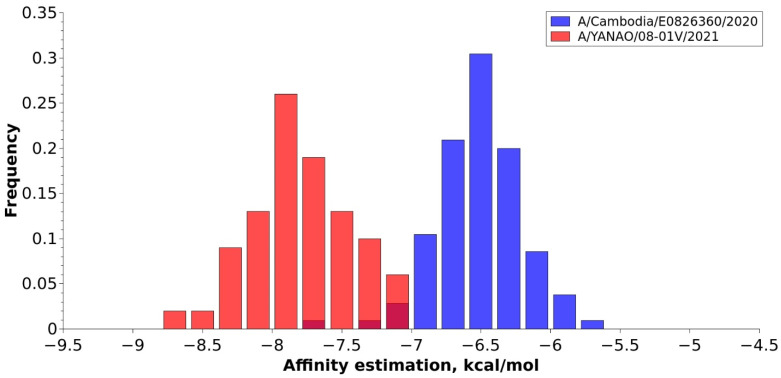
Affinity estimation results for HAs in the complex with the receptor analog of A/Cambodia/E0826360/2020 and A/YANAO/08-01V/2021. The height of each bar represents the relative occurrence frequency of structures corresponding to a certain score.

A molecular docking analysis of ligand-protein interactions of the studied HAs was performed to evaluate their affinity with Autodock Vina [22]. The results of the molecular docking estimations are presented in the form of a histogram in Figure 3 and expressed in kcal/mol values. The average docking score for A/YANAO/08-01V/2021 is 1.28 kcal/mol less than the A/Cambodia/E0826360/2020 average docking score. The standard deviations of the data are 0.35 kcal/mol and 0.31 kcal/mol for A/YANAO/08-01V/2021 and A/Cambodia/E0826360/2020, correspondingly. The lower docking score and molecular dynamics behavior of the receptor analog suggests that the A/YANAO/08-01V/2021 HA may bind to the terminal saccharides in the receptor more tightly.

### 3.5. In Vitro Receptor Specificity Analysis

Using the method of biolayer interferometry, several A(H3N2) influenza viruses were studied for receptor specificity to 6’-Sialyl-N-acetyllactosamine biotinylated receptor analogs. Among the studied viruses, there were two viruses from the 2a2 clade isolated in this study: A/YANAO/08-01V/2021 with a set of HA mutations (I25V, R201K, and S219Y) typical for H3N2 viruses which circulated in Russia in 2021–2022 and A/Yekaterinburg/10-01V/2021 without the three abovementioned mutations but with two other substitutions in HA (D53N and S156H). Additionally, we analyzed strains from the 2a1 comparison group (A/Cambodia/925256/2020 and A/Cambodia/e0826360/2020) and other reference viruses (A/Hong Kong/45/2019 (3C.2a1b.1b), A/Stockholm/5/2021 (2a2), and A/Darwin/09/2021 (CVV 2a2)). It was found that the strains of clade 2a2 had similar dissociation constants (K_D_) to the K_D_ of the reference strains (Table S1). 

## 4. Discussion 

### 4.1. Reemergence of Influenza Circulation in Russia and around the World in 2021–2022

The COVID-19 pandemic has significantly affected the circulation of seasonal influenza in Russia and around the world. The percentage of specimens positive for influenza viruses, among all specimens tested globally in the 2020–2021 epidemic season, dropped from 17% observed on average in 2017–2020 to less than 0.2% between September 2020 and February 2021 [8]. The drastic decrease in influenza circulation coincided with the implementation of measures against SAR-CoV-2 and global circulation of the virus, which may have led to viral interference in favor of SARS-CoV-2. 

Between September 2021 and February 2022, influenza A(H1N1)pdm09, A(H3N2), and influenza B viruses circulated the world in lower numbers than before the COVID-19 pandemic, but in greater numbers than during 2020–2021. Overall, the percentage of positive samples for influenza during this period was less than 3%, which was a significant level of circulation, but less than in similar reporting periods before the COVID-19 pandemic. Influenza A viruses predominated in Russia (mainly H3N2 according to FluNet), Europe, and America, and influenza B viruses circulated predominantly in China [10].

Influenza circulation in Russia and the world decreased by the end of January, which coincided with the peak of the SARS-CoV-2 Omicron wave (worldometers.info/coronavirus/country/russia/ (accessed on 1 September 2022), https://nextstrain.org/ (accessed on 1 September 2022)). The second wave of influenza, comparable to the level of circulation of the first, was observed in many countries of the Northern Hemisphere, starting in February 2022 with peak circulation in April and a decrease by June, and there was a global dominance of A(H3N2) FluNet [36,40]. The second wave of influenza virus circulation was not registered in Russia, possibly due to the reduction in tourist flows from Asia and Europe [FluNet]. The unprecedented absence of significant influenza circulation in the 2020–2021 season and the global resumption of influenza circulation in the 2021–2022 season raises a question about the reasons for this dynamic. 

The re-emergence of influenza circulation may have been made possible due to several factors, such as the easing of control measures for the COVID-19 pandemic in 2021, the emergence of herd immunity to SARS-CoV-2 due to the exposure of a large portion of the population to the virus via infection and due to large-scale vaccination, a decrease in immunity to seasonal influenza virus due to the very low levels of influenza circulation in the previous season, or possibly the emergence of a more adapted A(H3N2) strain that resulted in its global dominance [36,40]. Such dynamic in influenza seasons during the COVID-19 pandemic needs to be explored in more detail in order to evaluate the influence of a variety of factors (e.g., global travel, population immunity, and other epidemiological factors) on the seasonality of influenza, which is only partially understood [41]. 

### 4.2. Genetic and Virological Analysis of H3N2

All A(H3N2) viruses analyzed in Russia in the 2021–2022 season belong to the 3C.2a1b.2a.2 clade (Figure 1) and all have the HA mutations that characterize this group: the Y159N, T160I substitutions (which lead to the loss of the glycosylation site) and replacements for L164Q, G186D, and D190N. Analysis of Influenza A(H3N2) viruses isolated in Russia in 2021–2022 in HIAs demonstrated that 75% of analyzed A(H3N2) isolates were antigenically different from the 2021–2022 Northern Hemisphere vaccine A(H3N2) virus A/Cambodia/e0826360/2020 (clade 3C.2a1b.2a.1). At the same time, all tested A(H3N2) isolates (including viruses with mutations in HA I25V, R201K, and S219Y, common in Russia) were antigenically similar to both the egg-propagated 2022–2023 Northern Hemisphere vaccine A(H3N2) strain A/Darwin/9/2021 (clade 3C.2a1b.2a.2) and cell-propagated reference A(H3N2) virus A/Stockholm/5/2021 (clade 3C.2a1b.2a.2). All A(H3N2) isolates in Russia tested in HIA belonged to the clade 3C.2a1b.2a.2. This clade started its spread in North America and Europe in September 2021 when the strain A/Cambodia/e0826360/2020 (clade 3C.2a1b.2a.1) had already been included in the 2021–2022 Northern Hemisphere vaccine. A(H3N2) viruses of the 3C.2a1b.2a.2 clade dominated in circulation in the Northern Hemisphere in the 2021–2022 season. The hemagglutination inhibition assays performed by the WHO in collaborating centers on influenza in London and Atlanta showed that only a minority (7–17%) of H3N2 viruses tested in the 2021–2022 season were recognized well by the serum raised against the 2021–2022 egg-propagated vaccine strain A/Cambodia/e0826360/2020. At the same time, the majority (70–84%) of A(H3N2) viruses tested in the HIAs were antigenically similar to the egg-propagated strain A/Darwin/9/2021, which was recommended as a vaccine for the 2022–2023 Northern Hemisphere flu season [36,42,43].

### 4.3. Genetic Analysis of A(H1N1)pdm09

Influenza A(H1N1)pdm09 viruses circulated in many parts of the world, predominating in some regions, especially Africa, or occurring in small proportions or only sporadically. Viruses collected after January 2022 belonged to two subclades, 5a.1 and 5a.2. Viruses of subclade 5a.2 collected after January 2022 contained additional HA1 amino acid substitutions (K54Q, A186T, Q189E, E224A, R259K, and K308R) and the antigenic difference of viruses of this group detected by human serology was noted. Based on the data obtained, the WHO recommended the inclusion of a new strain, A/Sydney/5/2021 (H1N1)pdm09-like, which represents a newly identified group of A(H1N1)pdm09 viruses in subclade 5a2, in the vaccine for the Southern Hemisphere 2023 [36]. Vaccine effectiveness for the Northern Hemisphere for the 2022–2023 season against this group of A(H1N1)pdm09 viruses can be somewhat reduced. In the 2021–2022 season in Russia, only sporadic circulation of influenza A(H1N1)pdm09 was observed. One of the two A(H1N1)pdm09 viruses tested at the SRC VB “Vector” in the season 2021–2022 A/Khmao/182-14V/2021 (5a2) (20 December 2021) belonged to a new group of viruses of the 5a2 subclade. The rising dominance of the 5a2 group globally is expected based on the circulation dynamic, which shows a continuous increase in circulation of this group in 2022 based on analysis by the NextStrain of available genetic information in GISAID (Next Strain https://nextstrain.org/flu/seasonal/h1n1pdm/ha/2y, accessed on 1 September 2022). In the 2022–2023 season, there may be an increase in the circulation of the A(H1N1)pdm09 virus in Russia and the world due to the observed adaptive changes in the A(H1N1)pdm09 viruses and a decrease in heard immunity due to the very low circulation of A(H1N1)pdm09 in the 2020–2022 seasons.

### 4.4. Genetic and Virological Analysis of Type B Viruses

In sporadic circulation in Russia in the 2020–2021 season, two new subclades 3a1 and 3a2 of influenza B/Victoria virus were noted with a deletion of three amino acids in HA (position 162–164). Toward the end of the 2020–2021 season and in the 2021–2022 season in Russia, all detected B/Victoria viruses belonged to the 3a2 subclade.

During the 2021–2022 flu season, strains of the clade V1A.3a.2 were predominant among circulating B/Victoria viruses in the majority of regions worldwide [36]. The antigenic characterization of influenza B in our study correlated well with the results of the WHO CC in London; according to which, 92% of recently tested B/Victoria viruses of the V1A.3a.2 clade were antigenically different from the 2021–2022 Northern Hemisphere egg-propagated vaccine strain B/Washington/02/2019 (V1A.3). At the same time, the majority of V1A.3a.2 viruses were antigenically similar with the egg-propagated strain B/Austria/1359417/2021 included in the 2022–2023 Northern Hemisphere vaccine [43]. 

In 2020–2022, no B/Yamagata lineage viruses were detected in Russia. Since August 2020, there have been no confirmed B/Yamagata lineage viruses in the world for characterization [36]. 

All A(H3N2) and B/Victoria isolates that we tested in the neuraminidase inhibition assay were normally inhibited by neuraminidase inhibitors oseltamivir and zanamivir. The detection frequency of influenza viruses characterized by reduced or highly reduced inhibition by neuraminidase inhibitors remains low worldwide and does not exceed 1% of all tested strains [42,44]. Hence, neuraminidase inhibitors continue to be the first-line drugs for specific influenza treatment.

### 4.5. Population Immunity and Vaccine Effectiveness as Factors Associated with the Emergent and Dominant Circulation of A(H3N2) in the 2021–2022 Season

The dominant circulation of influenza A(H3N2) in Russia and other countries of the Northern Hemisphere, including Europe and the USA, was detected against the background of an antigenic difference between the circulating viruses of the 3C.2a1b.2a.2 clade and the vaccine strain for the Northern Hemisphere for the 2021–2022 season of the 3C.2a1b.2a.1 clade. Relatively low vaccine effectiveness of component A(H3N2) was noted in studies in the USA (35%) [45,46], Canada (36%), and Europe (35%) [47]. These estimated values of vaccine effectiveness were similar to previously reported values for the vaccine effectiveness study in 2009–2015 [48]. It is noted, that despite the relatively low vaccine effectiveness of the A(H3N2) vaccine component, vaccination remains an effective form of influenza control and can partially reduce the number of cases and their severity, as well as prevent complications. In Russia, prior to the 2020–2021 epidemic season, 85.9 million people, or 59% of the population, were vaccinated against influenza [49], on the eve of the 2021–2022 season, more than 69.1 million Russians were vaccinated, or 47.3% of the country’s population [50].

A study of herd immunity in Russia showed that the target level of 50% population seropositivity to vaccine influenza strains had not been achieved prior to the 2021–2022 epidemic season. This reveals the decrease in this indicator compared to previous years when the presence of immunity for seasonal influenza was detected among 50% to 68% of the population, depending on the region of Russia [51]. The low circulation of influenza viruses in 2020–2021 was likely a contributing factor in the decrease in population immunity to seasonal influenza in Russia.

An analysis of the effect of low influenza activity in the season of 2020–2021 on population immunity and the next influenza season made using data over multiple influenza seasons in the United States showed an expected significant increase in the burden of the disease and low population immunity in the case of continuous low influenza activity [52]. It was suggested that an increased level of vaccination is necessary for the compensation of the expected drop in herd immunity. 

The high incidence of childhood influenza infection observed in Russia in 2021–2022 may be partly due to the limitation in the formation of immunity in conditions of extremely low influenza circulation since May 2020; young children may not have been exposed to the influenza virus to develop immunity. The CDC, Atlanta also noted that lower levels of population immunity due to reduced virus circulation in the general population, and especially among young children, could lead to a wider spread of the disease and a potentially more serious epidemic if influenza virus circulation resumes. They also emphasized the importance of vaccination to control the incidence of influenza [53]. The high importance of vaccinations for protection against influenza in the Russian population is also noted by the Russian Federal Service for Surveillance on Consumer Rights Protection and Human Wellbeing, Rospotrebnadzor [54]. Vaccination will play a key role in maintaining population immunity in the face of fluctuating influenza circulation and, in addition, influenza virus immunity will help to protect the population from possible simultaneous or sequential infection with co-circulating influenza viruses and SARS-CoV-2.

### 4.6. Receptor Specificity of Circulating Clade 3C.2a1b.2a.2 A(H3N2) Viruses in Russia in 2021–2022

Previous studies indicate the occurrence of substantial changes in binding specificity and affinity in modern human H3N2 viruses. In particular, it was suggested that since 1968, the avidity of the A(H3N2) HA to human receptors has declined and may have resulted in the decreased epidemiological impact of A(H3N2) viruses [13]. 

The wide distribution of the A(H3N2) group 2a2 viruses in the world and their complete dominance in Russia in 2021–2022 demonstrated the ability of these viruses to spread successfully regardless of the SARS-CoV-2 co-circulation and pandemic mitigation measures. There could be multiple epidemiological, virological, and immunological reasons for this phenomenon, one of which could be associated with transmissibility. There are amino acid substitutions Y159N, T160I, and D190N in the HA of A(H3N2) group 2a2 viruses, which can potentially change receptor-binding properties based on previous studies [Flusurver] and as a result may affect transmissibility [12]. 

Molecular modeling methods were applied in this study for the comparative analysis of the receptor affinity of A(H3N2) clades 3C.2a1b.2a.2 (A/YANAO/08-01V/2021) and 3C.2a1b.2a.1 (A/Cambodia/e0826360/2020). 

Numerous studies show that carbohydrates are flexible and can populate a series of isomeric states in physiological conditions [55,56]. The conformational mobility of the receptor is an important object of study because the receptor topological profile impacts the receptor binding kinetics and plays a role in the specificity of HA-glycan recognition. Acquisition of images of the HA-receptor complexes with both high temporal and spatial resolution using an in vitro approach can be a highly complicated task. The molecular dynamics method is a convenient instrument for this purpose [56] and it is commonly used for HA-receptor conformational analysis [57,58]. The trisaccharide receptor analog, 6’-sialyl-N-acetyllactosamine, used in the study of the estimation of direct HA-receptor interactions is a standard ligand in crystallographic and molecular modeling research of hemagglutinin-receptor complexes [58,59,60]. The HA binding site consists of several key structural components including 130 and 220-loops and a 190-helix [61]. The receptor interacts with the binding site via multivalent weak interactions between the glycoprotein and receptor carbohydrate [62]. The analysis of the hemagglutinins using the molecular dynamics method showed a more rigid behavior of the receptor analog in the complex with A/YANAO/08-01V/2021 due to amino acid substitutions D190N and S186D in the 190-helix and S219Y in the 220-loop, which may indicate stronger binding and increased receptor affinity. The recent study of the evolution of the A(H3N2) HA receptor binding properties showed that the D225N substitution in HA had a key role in the reduction in the receptor binding ability among viruses that circulated during the 2005–2010 seasons [13]. At the same time, it is interesting to note that Asp is present again at this position in the HA of recently circulated viruses. Generally, the conformation changes that led to the reduction in receptor affinity were observed in the 220 loops since the year 1968. The substitution S219Y in the HA of A/YANAO/08-01V/2021 is located in the 220 loops and can significantly impact the loop conformation. The receptor analog in crystallographic data for the 2005 A(H3N2) HA was not fully resolved due to poor binding [13]; whereas, the structure was fully resolved for previously circulating A(H3N2) viruses in 1968 and 2004, indicating better binding of the receptor analog by their HAs and less mobility in the HAs binding site. This correlated with biolayer interferometry data that showed a decrease in receptor avidity in 2005 A(H3N2) [13]. In our study, the conformational changes of the receptor analog in the complex with the HA of A/Cambodia/E0826360/2020 are observed and are associated with the noticeable change of the two monosaccharide positions (Figures S1 and S2). Similar conformational changes also could have been a reason for the non-resolving of these monosaccharides’ structures in the crystallographic data for the 2005 HA; this behavior is consistent with the predicted lower affinity of the HA to the receptor. 

The molecular docking method was used to further analyze the receptor binding properties. The molecular docking method is commonly used for the research of ligand-protein interactions, prediction of binding affinities, and binding poses in drug development [63,64]. Autodock Vina [22] is a relatively new program for molecular docking combining Monte Carlo and gradient-based algorithms for the conformation search and a simple scoring function for interaction evaluations. Autodock suite is generally applied for the docking of carbohydrate molecules [32,55]. The accuracy of Autodock Vina predictions is comparable with another program from the Autodock suite [22]. We see the approach based on molecular docking as a perspective for the relative binding energy assessment of close structure systems in terms of accuracy-time efficiency, because other factors, besides interactions, often are not very important and do not give a significant contribution to the relative affinity difference for these systems. Application of the molecular docking method showed that the A/YANAO/08-01V/2021 HA has a lower docking score. This result together with molecular dynamics behavior suggests that the A/YANAO/08-01V/2021 HA may have higher human receptor affinity; essentially, it can lead to slower movements of the virions on the target cells. This can increase the probability of receptor activation and the beginning of endocytosis.

In vitro receptor specificity evaluation using the method of biolayer interferometry for the viruses of clade 3C.2a1b.2a.2, which circulated in Russia in 2021–2022 and including the virus A/YANAO/08-01V/2021, in comparison with viruses from clade 3C.2a1b.2a.1 and A/Hong Kong/45/2019 CVV (clade 3C.2a1b.1b) did not show differences in the dissociation constant when tested with a trisaccharide 6’-Sialyl-N-acetyllactosamine biotinylated receptor analog (Table S1). The values of the equilibrium dissociation constants for the studied strains were all of the same order of magnitude as the previously characterized strains [65,66]. However, it was previously shown that recent A(H3N2) viruses circulating since 2003 maintained binding to human-type receptors but bind with much higher affinity to receptors comprising branched glycans with extended poly-N-acetyl-lactosamine chains. Thus, it was suggested that using shorter glycans for the investigation of receptor affinity may not give an accurate picture of receptor specificity for A(H3N2) viruses [67]. Comparison of the receptor analog binding of A(H3N2) viruses from 2a1 and 2a2 clades showed that, similar to the results of the study using branched glycans [67], the 2a1 A(H3N2) had a good affinity to many a2,6-linked sialoside glycans with extended poly-LacNAc chains and/or branched structures, whereas the 2a2 A(H3N2) HA maintained strong binding mostly to an extended biantennary sialoside [14].

Thus, based on the molecular modeling data of this study, which indicates the possibility of stronger human receptor binding for new subgroup 2a2 viruses, it is necessary to conduct additional research to determine the affinity of binding of these strains in comparison to A(H3N2) strains of other clades to different variants of the human type of receptors, including extended biantennary sialoside and other a2,6-linked sialoside glycans with various extended poly-LacNAc chains. Such a study will provide an opportunity to investigate changes in receptor binding properties in the circulating A(H3N2).

In this study, the ensemble docking approach predicts possible adaptive changes in the receptor binding affinity of A(H3N2) viruses of the new and dominant clade 2a2, which may have contributed to increased infectivity and transmissibility and allowed these groups to outcompete the viruses of clade 2a1, overcome some of the anti-COVID-19 measures, and spread globally with dominance over large territories and many countries during SARS-CoV-2 co-circulation. Developed further and streamlined, the analysis of receptor specificity using molecular modeling may potentially become a powerful tool in assessing receptor specificity for influenza viruses based on hemagglutinin sequence and may contribute to epidemiological analysis and prognosis.

The constant emergence of new antigenic groups of A(H1N1)pdm09, A(H3N2), and influenza B viruses, possibly more adapted to transmission in the presence of quarantine measures and competition with SARS-CoV-2, requires constant careful monitoring and study of the properties of circulating influenza viruses in order to understand which strains can circulate and be more adaptive for epidemiological analysis and prognosis, and the selection of vaccine strains.

## 5. Conclusions 

The re-emergence of influenza circulation occurred in Russia in the 2021–2022 season in association with the lessening of anti-COVID-19 measures and the reduced immunity of the population to the influenza virus and an increase in population immunity to SARS-CoV-2. 

Clade 3C.2a1b.2a.2 A(H3N2) viruses dominated in Russia and the world in the 2021–2022 season and may be more adapted to transmission in the conditions of anti-COVID-19 measures and co-circulation with SARS-CoV-2. Molecular modeling predicted a higher affinity of the viruses to human upper respiratory tract receptors. Further investigation of the viruses is required for the evaluation of their possible adaptive advantages. 

Due to ongoing uncertainty surrounding the COVID-19 pandemic and its impact on influenza activity, it is critical to maintain ongoing global influenza surveillance and recommend influenza vaccination

## Supplementary Materials

The following supporting information can be downloaded at: https://www.mdpi.com/article/10.3390/pathogens11111388/s1, Figure S1: Time dependencies of atoms root mean square deviation; Figure S2: Several conformers of the receptor analog from the MD trajectories; Table S1: Receptor-binding assay using bio-layer interferometry.
