# Supplementary material for: Re-Emergence of Circulation of Seasonal Influenza during COVID-19 Pandemic in Russia and Receptor Specificity of New and Dominant Clade 3C.2a1b.2a.2 A(H3N2) Viruses in 2021–2022
Source: Pathogens. 2022 Nov 21;11(11):1388. doi: 10.3390/pathogens11111388 (PMC9698969; doi:10.3390/pathogens11111388)
Supplement: Supplementary file 1 [file pathogens-11-01388-s001.zip › Figure S1 Time dependencies of atoms root mean square deviation-1.pdf]

Figure S1: Time dependencies of atoms root mean square deviation

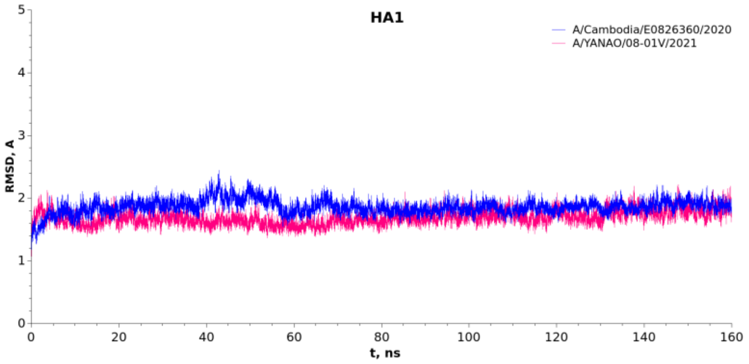

a

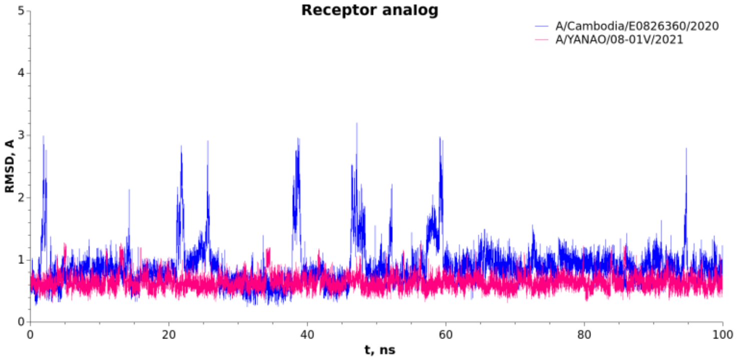

b

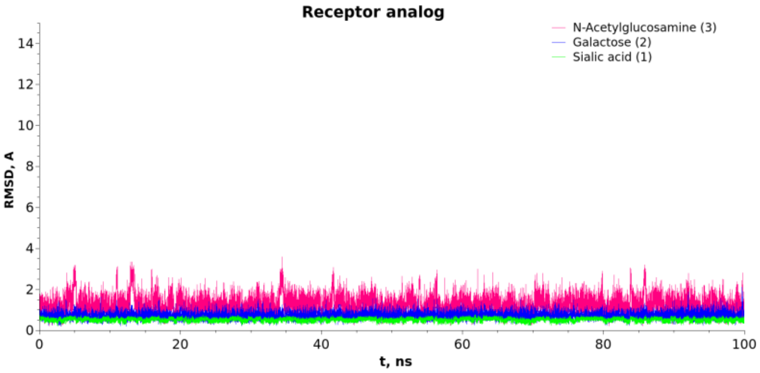

c

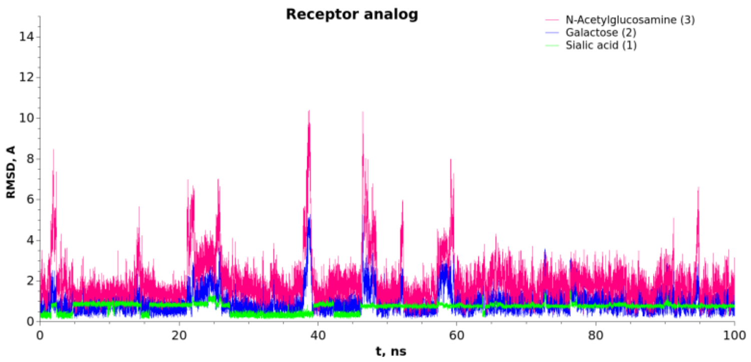

d

Root mean square deviations (a) of HA1 subunit bound to the receptor analog after the full simulation start; (b) of receptor analog after the equilibration process; per residue for the receptor analog with superimposing the sialic acid relatively to the first frame for the complexes with HAs of (c) A/YANAO/08-01V/2021; (d) A/Cambodia/e0826360/2020.

Generally, non-close to the crystallographic pose conformations of the receptor analog in complex with HA of A/Cambodia/e0826360/2020 are associated with cooperative changes in glycosidic torsion angles and the exposure of the N-Acetylglucosamine part of the molecule to solvent (Figure S2).
