# Supplementary material for: Re-Emergence of Circulation of Seasonal Influenza during COVID-19 Pandemic in Russia and Receptor Specificity of New and Dominant Clade 3C.2a1b.2a.2 A(H3N2) Viruses in 2021–2022
Source: Pathogens. 2022 Nov 21;11(11):1388. doi: 10.3390/pathogens11111388 (PMC9698969; doi:10.3390/pathogens11111388)
Supplement: Supplementary file 1 [file pathogens-11-01388-s001.zip › Figure S2 Several conformers of the receptor analog from the MD trajectories-1.pdf]

Figure S2: Several conformers of the receptor analog from the MD trajectories

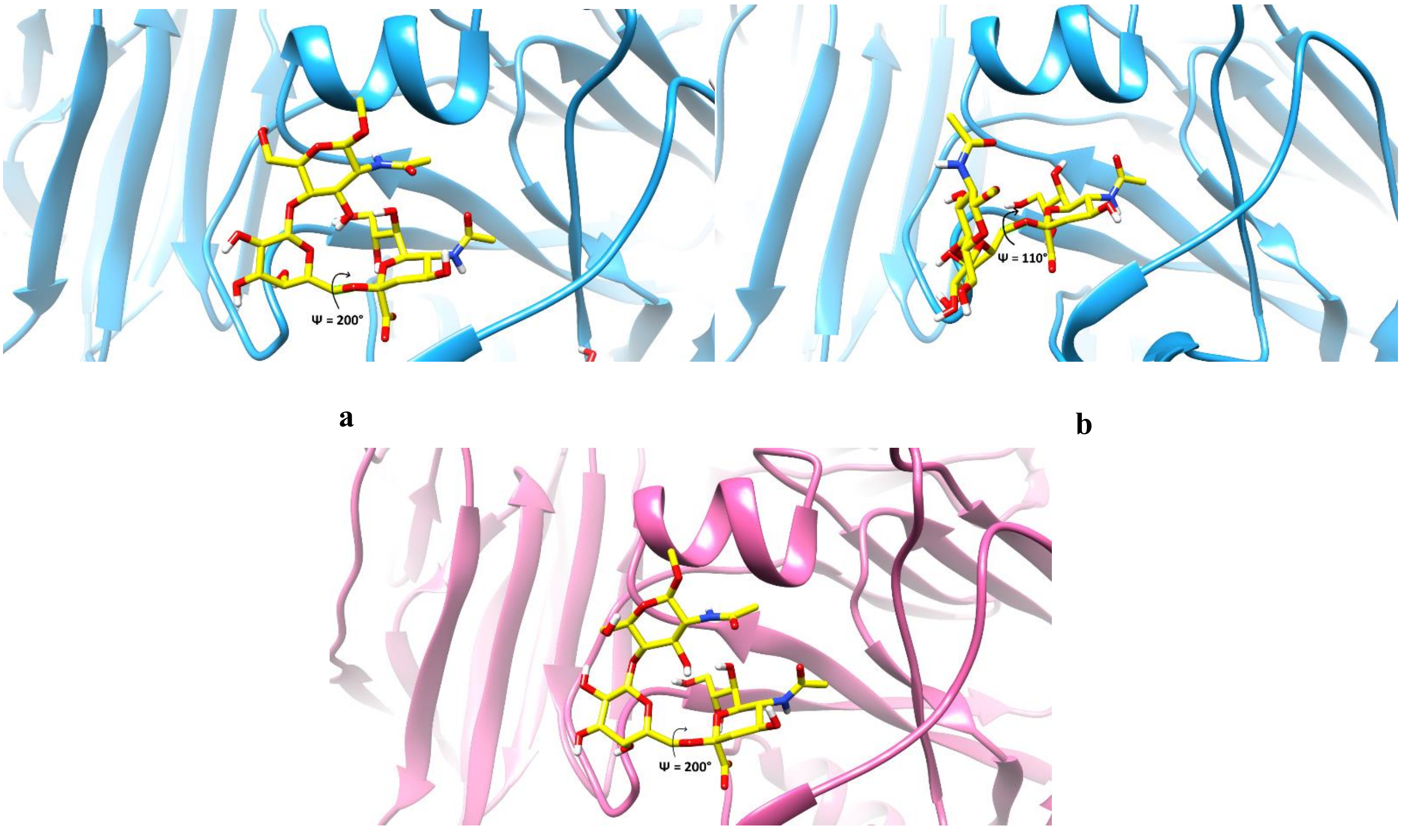

a b

c

(a) Conformer close to the crystallographic pose (b) conformer associated with the change of the glycosidic torsion angle Ψ by 90 degrees related to the complex with the HA of A/Cambodia/e0826360/2020; (c) conformer related to the complex with the HA of A/YANAO/08-01V/2021.

Receptor analog conformation kept close to the crystallographic pose during the full simulation time after the equilibration (Figure S1) for the complex with HA of A/YANAO/08-01V/2021.
