# Supplementary material for: Re-Emergence of Circulation of Seasonal Influenza during COVID-19 Pandemic in Russia and Receptor Specificity of New and Dominant Clade 3C.2a1b.2a.2 A(H3N2) Viruses in 2021–2022
Source: Pathogens. 2022 Nov 21;11(11):1388. doi: 10.3390/pathogens11111388 (PMC9698969; doi:10.3390/pathogens11111388)
Supplement: Supplementary file 1 [file pathogens-11-01388-s001.zip › Table S1.pdf]

Table S1. Receptor-binding assay using bio-layer interferometry (error does not exceed 3%).

Strain	K_D, µM	K_D, µM + 20 nM oseltamivir
A/Cambodia/925256/2020	0.002	0.01
A/Hong Kong/45/2019	0.01	0.01
A/Cambodia/e0826360/2020	0.04	0.01
A/Stockholm/5/2021	0.05	0.02
A/Yekaterinburg/10-01V/2021	0.005	0.02
A/YANAO/08-01V/2021	0.02	0.03
A/Darwin/09/2021	0.2	0.7
